# Fractionated Leaf Extracts of *Ocimum gratissimum* Inhibit the Proliferation and Induce Apoptosis of A549 Lung Adenocarcinoma Cells

**DOI:** 10.3390/nu16162737

**Published:** 2024-08-16

**Authors:** Rachael M. Curtis, Heng-Shan Wang, Xuan Luo, Erika B. Dugo, Jacqueline J. Stevens, Paul B. Tchounwou

**Affiliations:** 1 College of Science, Engineering, and Technology, Jackson State University, 1400 JR Lynch Street, Jackson, MS 39217, USA; jacqueline.j.stevens@jsums.edu (J.J.S.); paul.tchounwou@morgan.edu (P.B.T.); 2 School of Chemistry and Pharmacy, Guangxi Normal University, No. 15 Yu Cai Road, Guilin 541004, China; 3 Department of Applied Chemistry, School of Chemistry and Chemical Engineering, Guangxi University, No. 100 East Daxue Road, Nanning 530004, China; 4 Department of Natural and Behavioral Sciences, College of Science, Technology, Engineering, and Mathematics, Johnson C. Smith University, 100 Beatties Ford Road, Charlotte, NC 28216, USA; 5 RCMI Center for Urban Health Disparities Research and Innovation, Morgan State University, 1700 E. Cold Spring Lane, Baltimore, MD 21252, USA

**Keywords:** *Ocimum gratissimum*, A549 cells, apoptosis

## Abstract

Previous in vitro studies in our laboratory demonstrated that ethyl acetate (P_2_) and water- soluble (PS/PT1) fractionated leaf extracts of *Ocimum gratissimum* inhibit the proliferation of prostate cancer cells. It has been reported that the crude aqueous extract induces apoptosis in lung adenocarcinoma cells; however, the efficacy of the fractionated extracts against these cells remains unclear. In the present study, we hypothesized that the ability of the fractionated extracts to inhibit proliferation and induce apoptosis is associated with the activation of pro-apoptotic proteins and induction of DNA condensation in A549 cells. *Ocimum gratissimum* was cultivated and its leaves were harvested, extracted, and fractionated to produce fractions P_2_ and PS/PT1. Anti-proliferative activity was assessed by direct cell count. For morphological characterization of apoptosis, 4′,6-diamidino-2-phenylindole staining was employed. Western blot analysis was performed to evaluate the apoptotic activity of the fractionated extracts. In data generated from anti-proliferation studies, P_2_ significantly inhibited cell proliferation in a concentration-dependent manner; PS/PT1 elicited a decrease in the viability of cells, occurring at 500 µg/mL. 4′,6-diamidino-2-phenylindole staining revealed the induction of apoptosis, as evidenced by the formation of apoptotic bodies. Increased levels of pro-apoptotic proteins were observed as the concentrations of the fractionated extracts increased. These results suggest that fractionated leaf extracts of *Ocimum gratissimum* inhibit the proliferation and induce apoptosis of A549 cells.

## 1. Introduction

Lung cancer is the leading cause of cancer-related death worldwide [1]. Mainstream treatment options include surgery, radiation therapy, chemotherapy, or a combination of these treatments [2]. These expensive, harsh treatment options often produce toxic side effects in patients. Therefore, a critical need exists for the application of efficacious cancer treatments with low toxicity, which is commonly found in natural products.

Due to their abundant medicinal benefits, natural products are considered excellent sources for the discovery of novel anticancer agents. Cancer research has increasingly focused on using natural products such as vegetables, fruits, spices, and other plants to destroy cancer cells and improve overall health. Increasing evidence suggests that myricetin, a flavonoid found in fruits and vegetables, inhibits the migration of human lung adenocarcinoma cells [3]. Additionally, curcumin, the principal bioactive compound of the popular Indian spice, turmeric, has been shown to induce autophagy, and apoptosis, and inhibit invasion and proliferation in lung adenocarcinoma cells [4,5,6]. Furthermore, crocin, the major constituent of saffron, a spice derived from the flower of *Crocus sativus*, has been found to stimulate apoptosis and cell cycle arrest in lung adenocarcinoma cells [7]. These studies, amongst many, suggest that some compounds obtained from medicinal plants and herbs are excellent agents for the prevention and treatment of lung cancer.

The *Ocimum* genus belongs to the Lamiaceae family of aromatic mints, herbs, and shrubs that are native to tropical climates. The plants of this family possess unique pharmacological properties and they are often used as food and medicine. The extracts and essential oils of three well-known species in this family, *Ocimum basilicum*, *Ocimum sanctum*, and *Ocimum gratissimum*, are known to possess medicinal properties. Essential oils of *Ocimum basilicum* have demonstrated anti-fungal, anti-bacterial, and anti-oxidant capacity [8]. Specifically, when tested against liver carcinoma cells, ethanol leaf extracts of *O. bacilicum* were shown to possess free-radical scavenging and DNA protective properties, which may prevent carcinogenesis [9]. It has been reported that essential oils and ethanolic extracts from *O. sanctum* inhibit proliferation, trigger apoptosis, and impede angiogenesis in breast, prostate, and lung cancer cell lines [10,11,12].

The species of interest for this project, *Ocimum gratissimum* (*Og*), is a basil indigenous to West Africa [13]. Known as Clove Basil or Scent Leaf, *Og* has been used extensively in African traditional medicinal systems for centuries. In coastal Nigeria, the plant is used to treat respiratory infections, conjunctivitis, epilepsy, high fever, and diarrhea. *Og* has been shown to have anti-oxidant, anti-microbial, anti-fungal, anti-proliferative, anti-diarrheal, antiviral, anti-hyperglycemic, anti-anemic, anti-hyperlipidemic, dermal-protective, and cytotoxic properties that contribute to its pharmacological value [14,15,16,17,18,19,20,21]. There is anecdotal evidence that the leaf extracts of *Og* shrink hemorrhoids while obviating associated bleeding and itching. It is, therefore, not surprising that crude aqueous extracts of *Og* leaves are reported to also possess anti-inflammatory properties [22]. Terpenoids, alkaloids, flavonoids, saponins, steroids, tannins, anthraquinones, and cardiac glycosides are among several biologically active constituents said to be responsible for the activity of *Og* [23].

Previous studies conducted in our laboratory have shown that aqueous and fractionated leaf extracts of *Og* inhibit the proliferation of prostate cancer cells [24,25]. Within the past decade, the anti-cancer activity of extracts of *Og* has also been reported in liver, breast, osteosarcoma, and cervical cell lines [26,27,28,29]. Most notably, the aqueous extract of *Og* has been shown to activate both intrinsic and extrinsic apoptosis pathways in (A549) lung adenocarcinoma cells [30]; however, the effects of fractionated extracts of *Og* leaves against these cells have not been reported. Therefore, this study evaluated the efficacy of fractionated leaf extracts of *Ocimum gratissimum* (OGFEs) against human lung adenocarcinoma (A549) cells in vitro.

## 2. Materials and Methods

### 2.1. Plant Preparation

*Og* was cultivated in the Jackson State University greenhouse. The plants were grown for approximately three to four months and were then harvested by cutting off the stems. The leaves were picked from the stems and air-dried at room temperature for ten days. The leaves were turned daily to prevent mold growth and to increase drying capacity. A conventional blender was used to grind the leaves into a fine powder which was then sifted using a sieve to remove unwanted woody fibers. The powdered leaves were stored at 4 °C until ready for use.

### 2.2. Extract Preparation and Preparation of Stock Solution

*Og* was fractionated by Dr. Heng-Shan Wang (Guangxi Normal University, Guilin, China). Ethyl acetate soluble extract P_2_ was prepared as follows: 3 sets of 100 g of powdered *Og* leaves were extracted in 500 mL round-bottom flasks fitted with reflux condensers using 450 mL aliquots of 95% ethanol (EtOH) in a 50 °C water bath for 2 h. Extracts from all three flasks were vacuum-filtered, combined, diluted to 70% EtOH, and then incubated at −20 °C for 2 h to precipitate chlorophyll from the extract. The extract was vacuum-dried using a rotary evaporator. The extract was then completely re-dissolved in distilled water and acetone. The acetone was volatilized using the rotary evaporator at 50 °C with occasional rotation of the flask, yielding a dark brown crude ethanol extract. Using a 1 L separatory funnel, the crude ethanol extract was re-extracted with 250 mL ethyl acetate, collected, and then dried in a rotary evaporator, resulting in fraction P_2_. This was then stored at −20 °C until further use.

Dr. Xuan Luo (Guangxi University, Nanning, China) provided the water-soluble fraction, PS/PT1. The fraction was obtained by first extracting 500 g of powdered *Og* with distilled water three times at 50 °C. The water solutions were combined and dried using a freeze dryer (FD-1A-50, Beijing Boyikang Experiment Instrument Co. Ltd., Liangxiang Industrial Development Zone, Beijing, China). The freeze-dried extract was re-extracted with anhydrous methanol three times. Any remaining solid residue was then dissolved in distilled water and the solution was vacuum-filtered. Anhydrous ethanol was added to the filtrate to a final concentration of 40%. The solution was centrifuged at 4500 rpm for five minutes to yield the extract PS/PT1.

**Figure 1:**
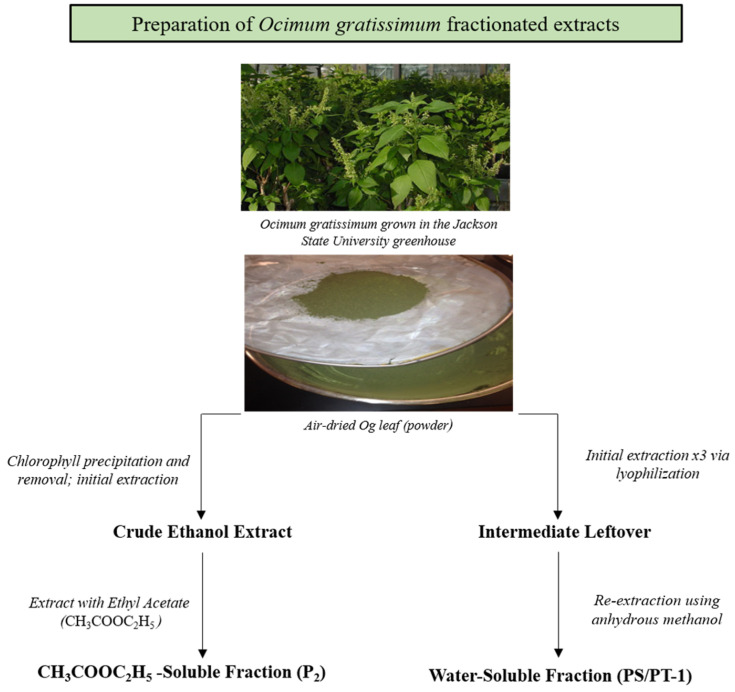
Preparation of *Ocimum gratissimum* fractionated extracts, P_2_ and PS/PT1.

The stock solutions were prepared as follows: P_2_ was dissolved in 0.8% EtOH, and PS/PT1 was dissolved in sterile distilled water (Figure 1). 

### 2.3. Cell Culture

A549 lung adenocarcinoma cells (Catalog #CCL-185) and F-12K medium were purchased from the American Type Culture Collection (Rockville, MD, USA). F-12K medium was supplemented with 10% fetal bovine serum (FBS) and 1% Penicillin/Streptomycin (Thermo Scientific, Waltham, MA, USA) to produce the complete growth medium (CGM).

A549 cells were cultured in Primaria™ tissue culture dishes, with dimensions of 100 × 20 mm, and maintained in a 5% CO_2_ humidified incubator at 37 °C. CGM was aspirated and refreshed every 48 h and the cells were grown until they were 100% confluent. Cell detachment was performed by incubating the cells with 0.25% trypsin (Thermo Scientific, Waltham, MA, USA). Detached cells were collected into a sterile centrifuge tube and cell density was calculated using a hemocytometer for subsequent analysis.

### 2.4. Anti-Proliferation Studies

Two milliliters of A549 cell suspension were seeded in Primaria™ 6-well tissue culture plates (BD Biosciences, San Jose, CA, USA) at a density of 5 × 10^4^ cells/mL. The cells were grown until they reached 65% confluency. Cells were treated with OGFEs at concentrations of 200, 300, 400, and 500 µg/mL and incubated again for 24 h. Cells grown in serum-free media served as the negative control and cells grown in CGM served as the positive control for testing with each fraction. For activity testing using P_2_, CGM was supplemented with the vehicle, 0.8% EtOH, which served as a second positive control. Cells were washed with 1 × PBS, harvested using a cell scraper, and collected into a sterile centrifuge tube. The density of viable cells was then calculated directly, using a hemocytometer.

### 2.5. 4′,6-Diamidino-2-Phenylindole (DAPI) Staining

DAPI staining was performed to observe chromatin condensation and nuclear morphological changes in A549 cells treated with *Og* extracts. Three milliliters of A549 cells were seeded in NUNC™ glass chamber slides (Lab Tek, Nunc Products, Naperville, IL, USA) at a density of 5 × 10^4^ cells/mL and grown to 65% confluency. The chamber slides were separated into two groups and were treated with 200 and 400 µg/mL OGFEs; one chamber slide remained untreated and served as the control. After 24 h of treatment, cells were washed with 1 × PBS and fixed to each slide by incubating with 10% trichloroacetic acid (MP Biomedicals, Solon, OH, USA) (10 min, 4 °C). The cells were washed thrice with ice-cold 1 × PBS before detaching the slide from the media chamber. Three drops of Prolong™ Gold Antifade reagent with DAPI solution (Invitrogen Corporation, Carlsbad, CA, USA) were added to the slide and allowed to cure for 24 h in the dark. Cells were visualized using an Olympus Epifluorescence Microscope (Center Valley, PA, USA) between 350 and 461 nm.

### 2.6. Western Immunoblot Analysis

For protein analysis, A549 cells were grown until approximately 90% confluent. Tissue culture dishes were separated into two groups and treated with OGFEs at concentrations of 200 and 400 µg/mL for 24 h; one dish remained untreated and served as the control. After incubation, cells were harvested and a pellet was obtained by centrifugation (2500 rpm, 5 min, 4 °C). The pellet was washed twice in ice-cold PBS, lysed in RIPA buffer, (20 mM Tris-HCl, pH 8.0, 0.5% (*w*/*v*) Nonidet P-40, 1 µg/mL leupeptin, 1.0 µg/mL pepstatin, 1 mM dithiothreitol, 1 mL PMSF, and 0.1 NaCl) vortexed, and then incubated on ice. The lysates were then clarified by centrifugation (13,500 rpm, 15 min, 4 °C). The supernatant was collected into sterile microcentrifuge tubes and protein concentration was determined using the Bicinchoninic Acid method. Cell lysates containing 100 µg of proteins were subjected to electrophoresis in a 10% SDS–polyacrylamide gel, using a BioRad^®^ Mini-PROTEAN Tetra Gel Electrophoresis System at 100 V (BioRad Corporation, Hercules, CA, USA). Protein was then slowly transferred to a polyvinylidene difluoride membrane by electroblotting overnight. The membrane was blocked with 5% (*w*/*v*) biotin-free non-fat dry milk and probed with monoclonal mouse anti-Caspase-8 (97446 s), anti-BH3 interacting-domain death agonist (BID) (8762), anti-Cytochrome C (12963), anti-Caspase-9 (9508 s), and anti-Caspase 3 (9668 s) (Cell Signaling, Technology, Danvers, MA) at a 1:1000 dilution overnight at 4 °C. The secondary antibody consisted of HRP-conjugated anti-mouse whole IgG used at a 1:10,000 dilution for 1 h at room temperature. Protein bands were then visualized using an enhanced chemiluminescence (ECL) detection system (GE Healthcare, Little Chalfont, Buckinghamshire, England).

### 2.7. Statistical Analysis

Quantitative data obtained from growth inhibition and DAPI staining experiments were presented as means ± SDs of triplicate counts. A *p* value < 0.05 was considered significantly different from the control according to Dunnett’s test.

## 3. Results

### 3.1. Determination of Anti-Proliferative Activity of OG Extracts on A549 Cells

**Figure 2:**
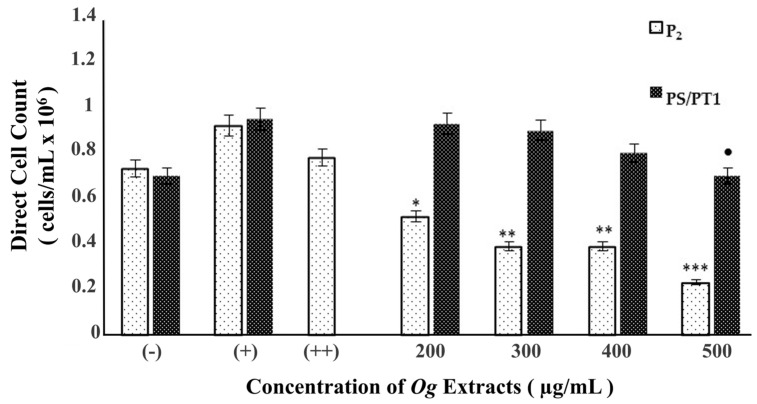
Determination of anti-proliferative activity of fractions P_2_ and PS/PT1 on A549 cells after 24 h of treatment. *p* values denote the level of significance of each treatment, when compared to (++, P2) and (+, PS/PT1), using Dunnett’s test. Corresponding *p* values: * = 0.003, ** = 0.0003, *** = 0.0007, ● = 0.01.

Figure 2 shows the results of the anti-proliferative activity of P_2_ and PS/PT1 OGFEs against A549 lung adenocarcinoma cells. The results represent the mean of triplicate counts ± SD. Exposure to all concentrations of P_2_ reduced cell proliferation in a concentration-dependent manner. Treatment with PS/PT1 resulted in a significant decrease in cell viability at 500 µg/mL only.

### 3.2. Determination of DNA Condensation and Morphological Changes in A549 Cells by Og Extracts, through DAPI Staining

**Figure 3:**
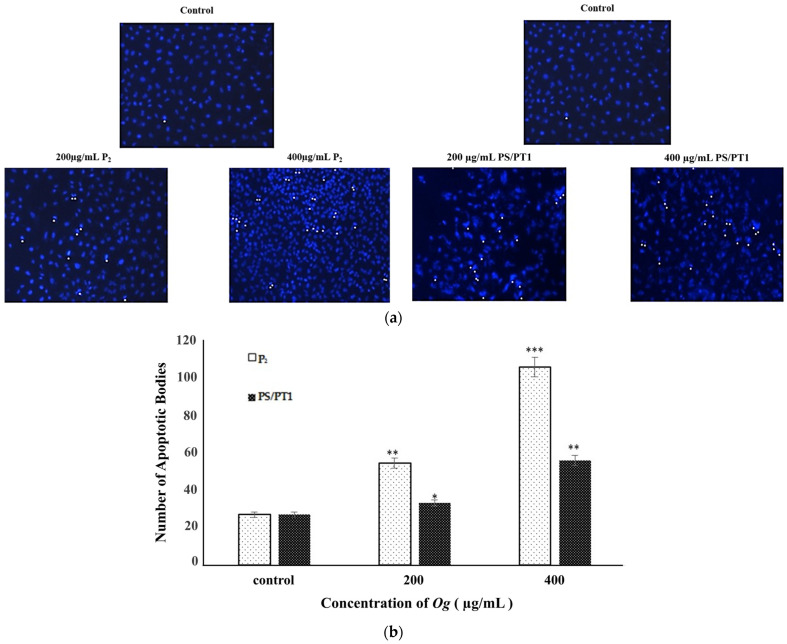
Determination of DNA condensation and nuclear morphological changes in A549 cells by fractions P_2_ and PS/PT1 through 4′,6-diamidino-2-phenylindole staining. (**a**) Represents photographed cells after treatment. Hyperfluorescence is indicated by white arrowheads. (**b**) Graphical representation of apoptotic body formation in cells from treatment with OGFEs. Corresponding *p* values: * = 0.05, ** = 0.003, *** = 0.0002.

To evaluate the ability of OGFEs to induce chromatin condensation and nuclear morphological changes in A549 cells, DAPI staining was performed. As shown in Figure 3a, viable cells displayed a normal nuclear size and fluorescence. Cells treated with OGFEs displayed hyper-fluorescence, characterized by the cell nuclei undergoing fragmentation and forming apoptotic bodies. In the treatment with both fractions, the number of apoptotic bodies was proportional to the treatment concentration (Table 1).

### 3.3. Assessment of Apoptotic Effects of Og Fractions by Western Immunoblot Analysis

**Figure 4:**
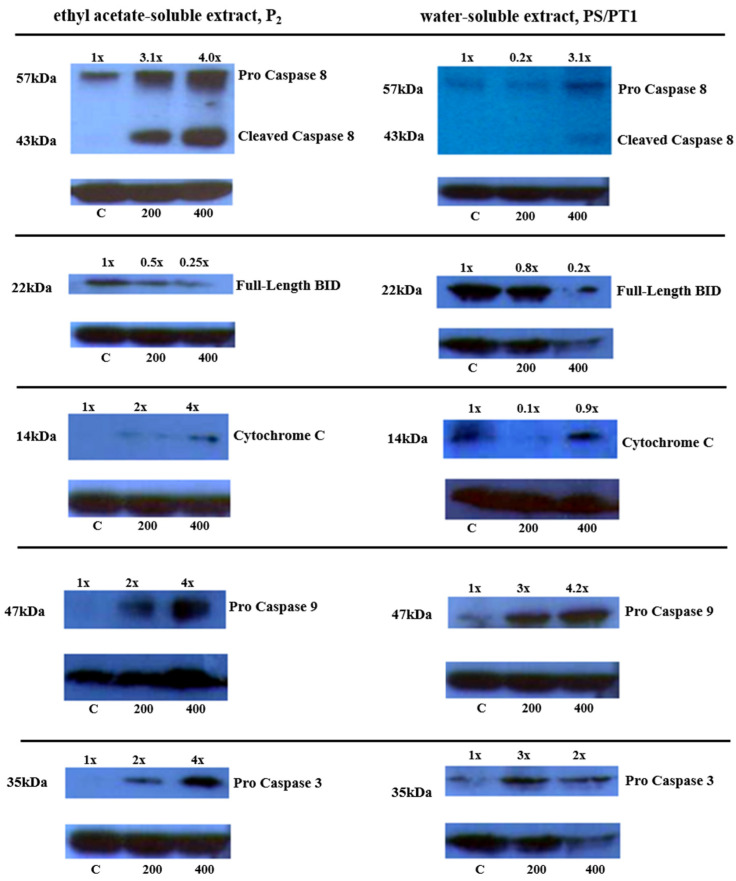
Evaluation of Caspase-8, BID, Cytochrome C, Caspase-9, and Caspase-3 proteins in A549 cells treated with fractions P_2_ and PS/PT1 for 24 h. C = untreated cells. 200 and 400 = cells treated with 200 and 400 µg/mL, respectively.

To evaluate the apoptotic activity of OGFEs, Western blot analysis was performed. A549 cells were treated with P_2_ and PS/PT1 extracts at concentrations of 200 µg/mL and 400 µg/mL for 24 h. Figure 4 displays the Western blots used to examine the levels of Caspase-8, BID, Cytochrome C, Caspase-9, and Caspase-3 proteins expressed in A549 cells treated with P_2_ and PS/PT1 fractions, respectively. Beta-actin was used as the loading control for all Western blot experiments.

A concentration-dependent increase in Caspase-8 expression was shown in cells treated with P_2_ and PS/PT1. Although P_2_ was found to have a stronger effect on the upregulation of Caspase-8 than PS/PT1 at 200 µg/mL and 400 µg/mL concentrations, both extracts were found to induce the production of cleaved Caspase-8. Decreased levels of full-length BID after exposure to P_2_ and PS/PT1 were also observed. Treatment with both extracts showed a noticeable degradation in this protein at 400 µg/mL. Both fractions were also found to increase the expression level of cytosolic Cytochrome C at 200 µg/mL and 400 µg/mL. Both P_2_ and PS/PT1 also increased the expression levels of Caspase 3 and Caspase-9.

## 4. Discussion

Lung cancer is the leading cause of global cancer incidence and mortality [31]. Although several studies in recent years have targeted potential therapies for the treatment of lung cancer, it remains a major health issue. The side effects that are associated with the established treatments for this disease can be toxic and debilitating for the patient, presenting a lack of selectivity for tumor cells, thereby negatively affecting healthy cells and tissues. Hence, there is a great need for less harsh, fortifying treatment agents, such as those harbored by plants and herbs.

Studies have shown that the extracts of various medicinal plants, bushes, and shrubs have profound effects on A549 cells [32,33]. It is believed that species within the *Ocimum* genus may qualify as potential candidates for the treatment of lung cancer, based on previous studies with various cancer cell lines [34,35,36,37]. Our laboratory has previously reported that fractionated extracts of *Ocimum gratssimum* leaf extract impede the growth of prostate cancer [24,25]. Furthermore, in A549 cells specifically, apoptotic signaling was induced by the crude aqueous extracts alone [30]. We previously determined the anti-proliferative activity of OGFEs to be significantly greater than that of the crude aqueous extract on prostate cancer cell lines [24]; therefore, we sought to determine if the same effect would occur in A549 cells. This study has now demonstrated that fractionated extracts more effectively decrease the proliferation of A549 cells than the aqueous extract previously reported [30]. We have also shown that the fractionated extracts of *Og* also activate apoptosis pathways to suppress cell viability in lung adenocarcinoma cells.

A549 cells were exposed to OGFESs to determine their effectiveness for growth inhibition. We observed that OGFEs inhibit the proliferation of A549 cells in a concentration-dependent manner. Although PS/PT1 hindered the growth of A549 cells at 500 ug/mL only, P_2_ had the strongest effect on growth inhibition, with a significant decrease in proliferation at all concentrations. Proliferation studies also determined the fractionated extracts to be more potent than the aqueous extract used in previous lung cancer studies [30], where significant cell death was observed at all concentrations using fraction P_2_ and at 500 µg/mL using fraction PS/PT1.

During apoptosis, there are morphological characteristics exhibited by cells. These may include detachment, cell shrinkage, chromatin condensation, and membrane blebbing [38]. DAPI permeates the cell membrane and yields blue fluorescence in viable cells; however, this fluorescence is heightened in the presence of apoptotic cells, as the dye penetrates the compromised membrane [39]. We examined the apoptotic features of A549 cells exposed to OGFEs. While viable cells displayed a normal nuclear size and fluorescence (Figure 3a), those treated with P_2_ and PS/PT1 exhibited hyperfluorescence, morphological alterations, and chromatin condensation in a concentration-dependent manner. Of all treatments, 400 µg/mL of fraction P_2_ produced the most apoptotic bodies.

The two best-known pathways of apoptosis are the intrinsic (mitochondrial) and extrinsic (death receptor-dependent) pathways. Both pathways are activated in response to stress and may lead to cell death through cytoskeleton cleavage [40]. The extrinsic apoptotic pathway is initiated by the binding of extracellular ligands to cell-surface death receptors. Through this pathway, Caspase-8 is activated and may also activate the downstream executioner, Caspase-3 [41]. Evidence suggests that Caspase 8 also plays a role in the intrinsic pathway through its interaction with BID [42]. Once cleaved by Caspase-8, BID trans-locates to the mitochondria and assists with the outpour of Cytochrome C. Once released into the cytosol, Cytochrome C binds with APAF-1 and Caspase-9 to form an apoptosome trimer. Caspase-9 is then cleaved from the complex to activate Caspase-3. Once activated, Caspase-3 cleaves the actin cytoskeleton, causing the cell to collapse [43,44]. The Western blot results of the present study indicate that exposure to OGFEs induces the upregulation of cleaved Caspase-8 expression. Whether this expression was stimulated via the intrinsic or extrinsic pathway is inconclusive; however, our findings confirm that BID was indeed stimulated by Caspase-8 cleavage. A concentration-dependent decrease in full-length BID was also observed. This decrease is attributable to it changing into its cleaved form, which translocates to the mitochondrial membrane to amplify the signal in the aforementioned apoptotic cascade. Ultimately, treatment with OGFEs leads to the activation of the Caspase-3 protein. Taken together, these findings indicate that fractions P_2_ and PS/PT1 upregulate the expression of pro-apoptotic proteins as a molecular mechanism for the inhibition of the proliferation of A549 cells.

## 5. Conclusions

The present study concludes that fractionated leaf extracts of *Ocimum gratissimum* inhibit the proliferation and strongly induce programmed cell death, as evidenced by DNA condensation, nuclear morphological changes, and upregulation of the expression of apoptotic proteins, in lung adenocarcinoma (A549) cells in a concentration-dependent manner. This is the first time that the effects of fractionated *Og* extracts have been evaluated on A549 cells. These results provide new insights into the therapeutic potential of *Og* and underscore the need for further pharmacological studies on the fractionation, isolation, purification, and testing of the anti-cancer activity of its active compounds.

## Figures and Tables

**Table 1:** Quantification of apoptotic bodies in A549 cells exposed to fractions P_2_ and PS/PT1. Data are expressed as means ± standard deviations.

Treatment	Mean Number of Apoptotic Bodies ± SD, Control	Mean Number of Apoptotic Bodies ± SD, 200 µg/mL	Mean Number of Apoptotic Bodies ± SD, 400 µg/mL
P_2_	27 ± 3.06	55 ± 4.04	107 ± 11.06
PS/PT1	27 ± 3.06	34 ± 4.16	57 ± 2.89

## Data Availability

The original contributions presented in the study are included in the article; further inquiries can be directed to the corresponding authors.
